# Associations between food deserts, food swamps, and ultra-processed food intake in childhood

**DOI:** 10.1590/1984-0462/2026/44/2025107

**Published:** 2026-05-11

**Authors:** Ana Clara da Cruz Della Torre, Bianca Araujo Milbratz, Wellington Segheto, Maysa Helena de Aguiar Toloni, Daniela Braga Lima

**Affiliations:** a Universidade Federal de Lavras, Departamento de Nutrição, Lavras, MG, Brazil.; b Universidade Federal de Alfenas, Faculdade de Nutrição, Alfenas, MG, Brazil.

**Keywords:** Food social space, Ultra-processed foods, Food consumption, Pediatric obesity, Espaço social alimentar, Alimentos ultraprocessados, Consumo alimentar, Obesidade pediátrica

## Abstract

**Objective:**

The aim of this study was to evaluate the association between retail food environments and consumption of ultra-processed foods in children.

**Methods:**

A cross-sectional study was conducted with 206 children aged 6–36 months who were followed up in primary health care. Consumption of ultra-processed foods was investigated using the intake marker form. Food deserts and swamps were identified using the Brazilian methodology proposed by the Food and Nutrition Security Intersectoral Chamber, and the density of healthy and unhealthy food outlets per 10,000 inhabitants was calculated. The associations between food environments and consumption of ultra-processed foods were estimated using binary logistic regression, yielding odds ratios (OR) and respective 95% confidence intervals (95%CI) from a generalized estimating equations model.

**Results:**

Ultra-processed foods were part of the diet in 60.18% of the children, with sweetened beverages being the most prevalent (42.22%). Living in sectors classified as food swamps was positively and independently associated with consumption of instant noodles, packaged snacks, or crackers (OR 2.71; 95%CI 1.19–6.16) and stuffed cookies/sweets/candies (OR 2.50; 95%CI 1.03–6.12).

**Conclusions:**

Children living in food swamps were more likely to consume ultra-processed foods. The results indicate the need to promote environments that favor healthy food choices among families and children through adequate nutrition, nutrition education, and policies that regulate access to and the advertising of unhealthy foods.

## RESUMO

**Objetivo:**

Avaliar a associação entre ambientes de varejo alimentar e consumo de alimentos ultraprocessados em crianças.

**Métodos:**

Estudo transversal com 206 crianças de 6 a 36 meses de idade acompanhadas na Atenção Primária à Saúde. O consumo de alimentos ultraprocessados foi investigado por meio do formulário de marcadores de ingestão. Desertos e pântanos alimentares foram identificados com base na metodologia brasileira proposta pela Câmara Intersetorial de Segurança Alimentar e Nutricional; ainda, calculou-se a densidade de estabelecimentos de alimentos saudáveis e não saudáveis por 10 mil habitantes. As associações entre ambientes alimentares e consumo de alimentos ultraprocessados foram estimadas por meio de regressão logística binária, apresentando *Odds Ratio* (OR) e respectivos Intervalos de Confiança de 95% (IC95%), com base no modelo de equações de estimativa generalizadas.

**Resultados:**

Alimentos ultraprocessados faziam parte da dieta de 60,18% das crianças, sendo as bebidas adoçadas as mais prevalentes (42,22%). Residir em setores classificados como pântanos alimentares associou-se positiva e independentemente ao consumo de macarrão instantâneo, salgadinhos de pacote ou biscoitos salgados (OR 2,71; IC95% 1,19–6,16) e biscoito recheado, doces ou guloseimas (OR 2,50; IC95% 1,03–6,12).

**Conclusões:**

As crianças que viviam em pântanos alimentares eram mais propensas a consumir alimentos ultraprocessados. Os resultados indicam a necessidade de promover ambientes que favoreçam escolhas alimentares saudáveis entre famílias e crianças, por meio de alimentação adequada, educação nutricional e políticas que regulem acesso e publicidade de alimentos não saudáveis.

## INTRODUCTION

The early years of life are a critical period marked by rapid growth and development, during which eating habits are established.^1^ Ensuring adequate health, food, and nutrition conditions is essential for optimal growth and long-term health.^2^ Brazilian^3^ and international^4^ guidelines recommend exclusive breastfeeding until 6 months, after which complementary foods should be introduced to meet nutritional needs.^2^ This stage is crucial for shaping eating behaviors, and dietary diversity contributes to healthy habits with lifelong effects.^5^

Over recent decades, global and Brazilian food environments have increasingly favored ultra-processed foods (UPFs) over natural or minimally processed options. This trend, driven by urbanization, evolving work routines, and the expansion of industrialized food systems, has made UPFs more available and accessible.^6,7^ In Brazil, their consumption rose between 2008 and 2018, especially among lower-income and less-educated groups.^8^ According to the Household Budget Survey (Pesquisaa de Orçamento Familiares 2017–2018), UPFs represent a substantial share of household diets.^9^ Among children, UPFs are present in the diet of 80.5% of those under 24 months and 93.0% of those over 24 months.^10^

Studies examining the influence of the food environment on eating habits have shown that this environment affects infant feeding.^10,11^ The food environment can be characterized by typologies: one is the “food swamp,” which describes areas with a high density of establishments that predominantly sell UPFs; and food deserts, characterized as neighborhoods, generally low-income, where access to healthy and nutritious foods is limited or non-existent.^12^ Some studies with school-age children^13^ and adolescents^14^ have shown that people living in neighborhoods with high availability of unhealthy food outlets are more susceptible to UPF consumption. On the other hand, research with children under 36 months old is still scarce in the literature.

This study is justified because early childhood represents a critical window for assessing growth and development, during which eating habits are formed and can influence health in both the short and long term by preventing chronic diseases.^1,5^ Understanding whether the food environment affects eating practices and nutritional status from the earliest stages is essential to identify harmful behaviors, guide nutritional interventions, and support public policies that promote health in this population. Additionally, the findings may inform the design of healthy environments that ensure food and nutrition security and promote well-being. This study aimed to investigate the association between residential exposure to food deserts and food swamps and children’s consumption of UPFs, hypothesizing that children living in food swamps are more likely to consume UPFs.

## METHOD

This study is a subset of a cross-sectional survey entitled “Children’s Health Booklet: Implications for food and nutritional security in early childhood”, which was conducted from March to October 2022 in a Brazilian municipality in southern Minas Gerais. The study was approved by the Ethics Committee (CAAE No. 43815221.2.0000.5148). Lavras is a university city with urban and agricultural activities, has 104,761 residents^15^ and a 77.9% rate of food insecurity among families with children aged 2–7, whose diets include many UPFs like stuffed biscuits, snacks, and petit Suisse cheese.^16^

**Figure 1:**
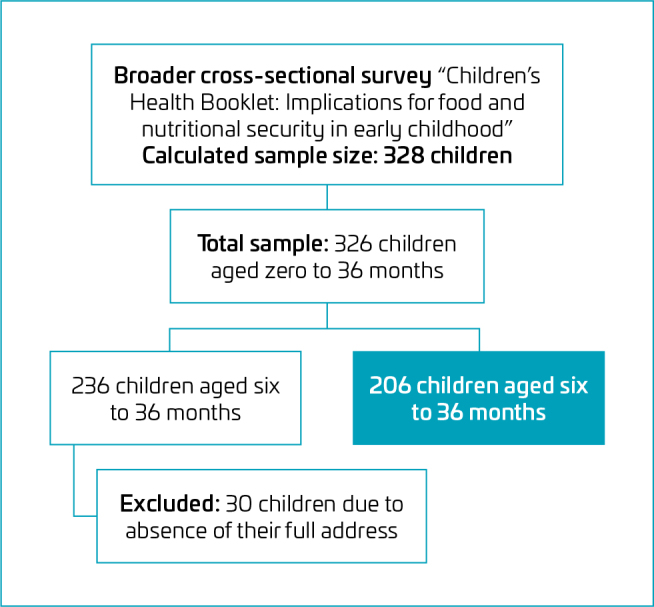
Study population and sampling flowchart.

Sample size calculation for the larger study was performed using the OpenEpi (3.01) software, based on the average number of live births in 2019 (1456), 2020 (1374), and 2021 (1309). Primary health care coverage in Lavras in 2022 was 67.07%.^17^ The formula for simple random samples of proportions was applied, assuming a population of children under 3 years of age comprising 2776 children (67.07% of total live births from 2019 to 2021), a 50% prevalence for multiple outcomes,^10^ a 95% confidence level, and a 5.2% margin of error, resulting in an estimated sample size of 356 children. Considering that the target population of children born in Lavras between 2019 and 2021 was finite, a finite population correction was applied, yielding a final required sample of 315 children. However, data were ultimately collected from 324 children aged 0–36 months. For this study, only a subsample of children aged 6–36 months was included, after excluding 90 children who were exclusively breastfed and another 30 due to missing or incomplete address information, resulting in a final sample of 206 children (Figure 1).

Children with clinical conditions or immobilizations that prevented anthropometric measurements, and those with incomplete or missing residential addresses, were excluded because geocoding was required for analyses related to the food environment. Data collection was carried out at 19 Family Health Strategy (FHS) units. Participants were approached during pediatric care appointments and through home visits. Interviewers explained the study objectives and procedures to each parent–child dyad, and data collection began only after written informed consent was obtained. According to records of care from the 19 FHS units in Lavras, Minas Gerais, 2797 children received care in 2022, and during the collection period, 2117 children aged 0–36 months received care.

The UPF consumption the previous day, namely: processed meats, sweetened beverages, instant noodles/salty snacks/crackers, and stuffed cookies/sweets/candies. To this end, we resorted to direct questions (Yes/No/I don’t know) that referred to UPF consumption from the food intake marker forms for children aged between 6 and 23 months old and those aged at least 2 years old developed by the Brazilian Food and Nutrition Surveillance System^18^ for children aged 6–23 months. For children older than 23 months, the form was adapted to capture consumption patterns appropriate for this age group while maintaining comparability with younger children.^18^

The independent variables included parents’ characteristics such as gender (female/male); maternal age (≤30 years old, >30 years old); skin color (white/non-white); marital status (with partner/without partner); schooling level (≥12 years of study/<12 years of study); work (paid/unpaid); nutritional status, assessed by means of the body mass index (BMI) and following the protocol adopted by the Ministry of Health (not excess weight/excess weight);^19^ and consumption of at least one UPF the day before the survey (no/yes)^18^ Some household characteristics were investigated, such as total family income (>2 minimum wages/≤2 minimum wages) and food insecurity risk (no/yes), defined based on applying the Screening for Food Insecurity Risk in Primary Health Care.^20^

Data on the retail food environment were obtained from Municipal Health Surveillance Department databases, including the following variables: the outlet’s municipal code, address, corporate name, National Registry of Legal Entities (*Cadastro Nacional da Pessoa Jurídica*, CNPJ), and National Classification of Economic Activities (*Classificação Nacional de Atividades Econômicas*, CNAE) corresponding to 17 types of outlets in the municipality. In addition, Public Outlets for Food and Nutrition Security (*Estabelecimentos Públicos de Segurança Alimentar E Nutricional*, EPSAN) were included. Geographic coordinates (latitude and longitude) of all outlets were retrieved using Google Earth Pro. The food outlets were classified according to the Technical Study for the Mapping of Food Deserts in Brazil.^21^ They were grouped into three categories: outlets mainly selling unprocessed or minimally processed foods; mixed outlets (the ones selling fresh or minimally-processed and ultra-processed food); and outlets selling UPFs.

Census tracts served as geographic units to analyze the retail food environment and the surroundings of participating families. Our analysis included children from 50 tracts (39.0% of georeferenced areas). Participants’ residences and food outlets were georeferenced by address, and geographic coordinates were obtained using QGIS 2.10.1. Food deserts were identified by calculating the density of healthy stores (retailers predominantly selling unprocessed or minimally processed foods and mixed outlets) per 10,000 inhabitants.^21^ The food deserts were defined as census tracts with a density of stores selling unprocessed or minimally processed foods and mixed outlets below or equal to the 25th percentile of the distribution in all tracts.^21^ Food swamps were identified by calculating the density of outlets that mainly sell UPFs.^6^ These swamps were defined as areas with UPF retailers above the 25th percentile.^6^

Tie data were double-entered into EpiInfo^®^ for subsequent consistency analysis. Frequency distributions, central tendency, and dispersion measures were estimated in the descriptive data analysis. Associations between food consumption and independent variables were examined using logistic regression with Generalized Estimation Equation (GEE) models and an exchangeable correlation matrix. Odds ratios (ORs) and 95% confidence intervals (CIs) were calculated, and variable collinearity and the Quasi-likelihood under the Independence model Criterion were assessed. Models were adjusted for mother’s age;^1,2,5^ family income;^9,16^ schooling;^1,2^ skin color;^6^ employment situation;^1,22^ BMI;^23,24^ food insecurity risk;^16,20^ and UPF consumption by the parents or guardians on the previous day,^22,23^ based on prior evidence linking these factors to children’s dietary patterns and nutritional risk. A backward elimination procedure was then applied at each model level, removing variables not associated at the 5% significance level. Analyses were performed using Stata (version 13.1), with a 5% significance level adopted throughout.

## RESULTS

Data from 206 children were analyzed. Most were female, over 12 months old, non-White, with adequate birth weight (>2500 g), born by cesarean section, not overweight, and breastfed within the first hour of life. Regarding UPF consumption, 60.19% (n=124) consumed at least one of the four food groups, with sweetened beverages showing the highest prevalence (42.22%; n=87), while only 2.91% (n=6) consumed all UPF types (Table 1). Overall, 75.73% (n=156) of children and their parents lived in food swamps (Table 2). Among them, 80.13% of parents were non-White, 62.99% earned <2 minimum wages, and 83.33% were overweight. In food deserts, 69.23% of parents were non-White, 69.24% had not completed high school, and 64.10% were overweight (data not shown).

**Table 1:** Demographic, birth, anthropometric, and food consumption characteristics of children aged between 6 and 36 months, Lavras (MG), 2022.

Variables	n	%
Sex		
Female	110	53.40
Male	96	46.60
Age (months)		
6–12	67	32.52
>12–24	99	48.06
>24	40	19.42
Color or race		
White	61	29.61
Non-white	145	70.39
Birth weight^*^		
<2500 g	26	12.87
>2500 g	176	87.13
Childbirth		
Natural childbirth	95	46.12
Cesarean section	111	53.88
Breastfeeding in the first hour of life^†^		
Yes	143	70.44
No	60	29.56
Nutritional status		
Not overweight	142	68.93
Overweight	64	31.07
Previous day’s UPF consumption		
No consumption	82	39.81
1 UPF	53	25.73
2 UPF	39	18.93
3 UPF	26	12.62
4 UPF	6	2.91

*n=202;

^†^n=203.

n: absolute frequency; %: relative frequency; UPF: ultra-processed foods.

Source: The authors; 2024.

**Table 2:** Characteristics of the sample and the food environment with the odds ratios and 95% confidence intervals of the binary logistic regression for the consumption of ultra-processed foods in children aged between 6 and 36 months, Lavras (MG), 2022.

Variables	Total		Processed meats			Sweetened beverages			Instant noodles/salty snacks/crackers			Stuffed cookies/sweets/candies		
	n	(%)	Yes (%)	No (%)	OR (95%CI)	Yes (%)	No (%)	OR (95%CI)	Yes (%)	No (%)	OR (95%CI)	Yes (%)	No (%)	OR (95%CI)
Gender (Male)	12	5.8	6.2	5.8	1.03 (0.12–9.33)	4.6	6.7	0.56 (0.16–1.97)	6.4	5.6	1.09 (0.31–3.81)	4.4	6.5	0.67 (0.17–2.54)
Mother’s age >30 years	70	34.1	35.4	18.7	0.38^*^ (0.09–1.52)	39.8	26.4	0.51^†^ (0.28–0.95)	37.5	26.2	0.61^*^ (0.32–1.15)	37.7	26.9	0.41^*^ (0.10–1.68)
Non-white skin color	161	78.2	81.2	77.9	1.25 (0.36–4.72)	82.8	74.8	1.27 (0.67–2.44)	90.3	72.9	3.44^†^ (1.35–8.74)	83.8	75.4	1.75^*^ (0.83–3.71)
Living without a partner^‡^	114	57.0	56.5	62.5	1.28 (0.44–3.70)	51.2	61.0	0.72 (0.41–1.25)	51.7	59.3	0.76 (0.42–1.41)	60.0	55.6	1.17 (0.64–2.14)
Education <12 years	112	54.4	43.7	55.3	0.63 (0.22–1.77)	64.4	47.1	2.11^†^ (1.19–3.73)	64.5	50.0	1.83^*^ (0.98–3.40)	55.9	53.6	1.09 (0.61–1.96)
Family income^§^ ≤2 minimum wages	126	62.4	35.7	64.4	0.31^†^ (0.10–0.98)	67.9	58.5	1.75^*^ (1.00–3.07)	62.7	62.2	0.99 (0.53–1.87)	47.7	69.3	0.40^†^ (0.22–0.74)
Unpaid word	96	47.5	43.7	47.8	0.85 (0.29–2.41)	54.0	42.6	1.88^†^ (1.13–3.14)	58.1	42.2	1.85^†^ (1.01–3.41)	44.8	48.9	0.85 (0.48–1.52)
Overweight^//^	117	57.3	56.2	57.4	0.98 (0.34–2.81)	56.5	58.0	1.01 (0.58–1.76)	51.6	59.9	0.73 (0.40–1.34)	50.7	60.6	0.67^*^ (0.37–1.21)
Risk of food insecurity	88	42.7	31.2	43.7	0.57 (0.18–1.76)	48.2	38.7	1.38^†^ (0.79–2.42)	53.2	38.2	1.83^*^ (1.00–3.35)	38.2	44.9	0.76 (0.42–1.37)
UPF consumption on the previous day	175	84.9	93.7	84.2	2.83 (0.35–22.6)	95.4	77.3	6.65^†^ (2.47–17.9)	95.2	95.4	5.34^†^ (1.45–19.6)	80.4	94.1	3.89^†^ (1.30–11.6)
Food desert	39	18.9	25.0	18.4	1.51 (0.48–4.77)	20.7	17.6	1.26 (0.69–2.33)	11.3	22.2	0.47^*^ (0.18–1.17)	14.7	21.0	0.63 (0.29–1.37)
Food swamp	156	75.7	81.2	75.3	1.30 (0.38–4.41)	80.5	72.3	1.12 (0.69–1.83)	85.9	71.5	2.37^†^ (1.08–5.21)	83.8	71.7	2.10^*^ (0.97–4.51)

*p<0.20

^†^p<0.05;

^‡^n=200;

^§^n=202;

^//^n=204.

OR: odds ratio; 95%CI: 95% confidence interval.

Source: The authors; 2024.

Earning less than two minimum wages was associated with children’s consumption of UPFs (OR 0.31, 95%CI 0.10–0.80). Consumption of sweetened beverages was associated with maternal age (OR 0.51; 95%CI 0.28–0.95), caregivers’ schooling level (OR 2.11, 95%CI 1.19–3.73), no paid work (OR 1.88, 95%CI 1.13–3.14), and UPF consumption by the parents (OR 6.65; 95%CI 2.47–17.93) (Table 2).

Living in a food swamp increased the odds of children consuming instant noodles/salty snacks/crackers (OR 2.37; 95%CI 1.08–5.21). Parental factors associated with this outcome included non-White skin color (OR 3.44; 95%CI 1.35–8.74), unemployment (OR 1.85; 95%CI 1.01–3.41), and UPF consumption (OR 5.34; 95%CI 1.45–19.61). For stuffed cookies/sweets/candies, household income ≤2 minimum wages was inversely associated (OR 0.40; 95%CI 0.22–0.74), while caregivers’ UPF consumption increased the odds (OR 3.89; 95%CI 1.30–11.62) (Table 2).

**Figure 2:**
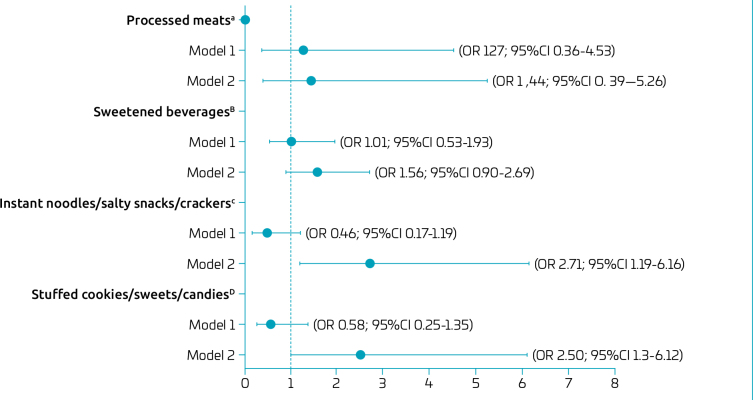
Binary logistic regression (reference category, non-food desert and non-food swamp for model 1 and model 2, respectively) for the food consumption of ultra-processed foods in children aged between six and 36 months, Lavras (MG), 2022.

^a^adjusted for mother’s age and family income; ^b^adjusted for mother’s age, family income, parent’s education and work, and ultra-processed foods consumption by the caregiver on the previous day; ^c^adjusted for mother’s age, colour or race, education and work of the caregiver, risk of food insecurity, and ultra-processed foods consumption by the caregiver on the previous day; ^d^adjusted for mother’s age, colour or race, body mass index of the caregiver, family income, and ultra-processed foods consumption by the caregiver on the previous day. OR: odds ratio; 95%CI: 95% confidence interval.

After adjustment, living in a food swamp remained positively associated with consumption of instant noodles/salty snacks/crackers (OR 2.71; 95%CI 1.19–6.16) and stuffed cookies/sweets/candies (OR 2.50; 95%CI 1.03–6.12) (Figure 2).

## DISCUSSION

The results indicate a high prevalence of UPF consumption among children, with about 60% reporting intake the day before the interview. In univariate analysis, children from households earning above two minimum wages had lower odds of consuming processed meats and stuffed cookies, sweets, or candies. Similarly, maternal age over 30 was associated with reduced odds of consuming sugar-sweetened beverages. Conversely, higher odds of consuming sugar-sweetened beverages were observed among children whose caregivers had low education, were unemployed, and reported UPF intake the previous day. Consumption of instant noodles, salty snacks, or crackers was more likely among children whose caregivers were unemployed, did not self-identify as White, reported prior-day UPF intake, and lived in food swamps. Caregiver UPF consumption was also associated with higher odds of children consuming stuffed cookies, sweets, or candies. Finally, sugar-sweetened beverage intake was associated with higher odds of consuming instant noodles, snacks, crackers, stuffed cookies, sweets, or candies.

UPF consumption was observed across all age groups, with UPFs introduced from early childhood,^25^ as observed in our study. These data may reflect the change in the dietary pattern of the Brazilian population observed over the years. There has been a systematic increase in the purchase and availability of UPFs in Brazilian households, making them increasingly present in the diet, while the proportion of UPFs, culinary ingredients, and processed foods has decreased.^9^ In addition, UPFs accounted for 19.70% of the calories available in households, reflecting a 1.02% increase; this increase in UPF consumption suggests a significant change in the population’s eating habits,^8^ which may have been reflected in our findings.

Findings showed that three out of four children lived in food swamps, which may explain the higher UPF consumption — especially instant noodles/salty snacks/crackers, stuffed cookies, sweets, or candies — among residents of these areas. Food swamps have more outlets selling unhealthy than healthy foods.^12^ In Brazil, a study with children under 10 years found that greater UPF availability in local outlets increased UPF intake and reduced consumption of minimally processed foods.^13^ In the United States, food swamps were identified as stronger predictors of adult obesity rates than food deserts.^26^ On the other hand, a randomized study conducted in the Lower Mississippi Delta with 150 mother-child dyads found no significant association between living in food swamps and poorer diet quality. However, participants living in these areas scored lower on diet quality, particularly in terms of empty-calorie intake.^27^ Therefore, although these previous studies were not conducted with children in the same age group as the current research, which may be a determining factor regarding dietary patterns, our data indicate the possibility that the children’s parents are subject to the influence of higher UPF availability in their area of residence, which eases the supply and consumption of these foods in their homes.

In Brazil, the presence of unhealthy food outlets is linked to lower consumption of healthy foods.^28^ The surrounding food environment likely encourages families to buy and store unhealthy products, making them easily accessible to children, who lack autonomy over food choices. Eating habits formed during growth influence lifelong dietary patterns and the risk of chronic diseases.^5^ Therefore, primary caregivers play a key role in shaping a home food environment that prioritizes access to healthy foods, particularly at this stage of life.

Although no association was observed after adjusting for parents’ UPF consumption based on their children’s UPF intake, the literature indicates that parents’ food consumption can influence their children’s food choices.^29^ The process of learning and shaping eating habits and preferences in children is based on repetition: children will reproduce their parents’ behaviors. Therefore, parents are the main influencers of a child’s diet.^23^

Brazilian secondary data revealed that the regular consumption of sugary drinks by adults living in the household was a risk factor for their children under 2 years of age also consuming them.^22^ This demonstrates that parental UPF consumption can stimulate similar behaviors in their children, which was also observed in our study.^22^

UPFs are commonly marketed to the public through strategies that imply they are safe and well-composed food options, especially for children. These products are readily available in several regions and outlets throughout Brazil, including traditional peoples and communities. This accessibility eases the incorporation of these foods into family diets and, consequently, can lead to nutritional disorders^30^ and adverse health outcomes.^24^

Recently, a Brazilian study reported an 80.50% prevalence of UPF consumption among children aged 6–23 months, with a 93.0% prevalence of sweetened beverages as the most consumed products among children aged 24–59 months.^10^ Our data reflect this trend of high UPF consumption among this age group nationwide.

Curiously, our data found that processed meat consumption was inversely associated with family income of less than 2 years, while children’s consumption of these UPFs was significantly associated with this. This finding contrasts with evidence from the literature,^8^ which indicates a reduction in consumption of these foods among individuals with higher levels of education and those in the highest income quintile. This divergence may reflect regional specificities, recent changes in dietary patterns, or even marketing strategies targeting lower-income populations. Factors such as limited access to natural foods, preparation time, and advertising may contribute to the pattern observed in our study.

The results should be interpreted with caution due to limitations related to self-reported data, cross-sectional design, and the food consumption marker, which assesses only the previous day’s intake and does not quantify nutrients or identify specific items (e.g., pizza, fried foods, dairy drinks). Exclusively breastfed children under 6 months were excluded due to instrument constraints. Georeferencing may introduce measurement and classification errors, and GEE models with small samples and multiple covariates may yield less precise estimates. Additionally, Basis Health Unit follow-up data were available only for children aged 0–36 months, without age stratification, which may affect generalizability. Nonetheless, the instrument is brief, easy to apply, and aligned with the Brazilian Food Guide,^18^ enabling robust analysis of the relationship between food environment and UPF consumption in early childhood, addressing key gaps in the literature.

The results of this study highlight the need for coordinated action across different sectors, articulating regulatory, urban, and educational measures. Zoning laws should be adopted to restrict the concentration and access of establishments selling unhealthy foods, while simultaneously encouraging fiscal and structural policies to promote the establishment of healthy food retailers, prioritizing neighborhoods with greater socioeconomic vulnerability.^14,26^ At the same time, it is imperative to invest in ongoing, evidence-based educational strategies to promote appropriate and healthy eating practices among children and their families as an essential component of a sustainable approach to reducing UPF consumption and improving child health.

In summary, children living in food swamps were more likely to consume UPFs, which harm health in both the short and long term. This study deepens the understanding of dietary determinants and supports the development of public actions and policies promoting healthy nutrition through improved food environments and sustainable urban planning. The findings underscore the need for intersectoral policies integrating territorial regulation and education within PHC to guide families on early-life feeding, timely food introduction, and reduced UPF exposure. Longitudinal studies are needed to better understand environmental influences on children’s diet and nutrition, as well as research on how perceptions of food and home environments affect consumption among children who do not buy food themselves.

In conclusion, children living in food swamps were more likely to consume UPFs. The results indicate the need to promote environments that favor healthy food choices among families and children, through adequate nutrition, nutrition education, and policies that regulate access to and the advertising of unhealthy foods.

## Data Availability

The database that originated the article is available with the corresponding author.
